# The role of magnetic resonance imaging in the diagnosis and prognosis of dementia

**DOI:** 10.17305/bjbms.2022.8085

**Published:** 2023-03-16

**Authors:** Milica Živanović, Aleksandra Aracki Trenkić, Vuk Milošević, Dragan Stojanov, Miroslav Mišić, Milica Radovanović, Vukota Radovanović

**Affiliations:** 1 Center for Radiology, University Clinical Center Niš, Niš, Serbia; 2 Faculty of Medicine, University of Niš, Niš, Serbia; 3 Clinic of Neurology, University Clinical Center Niš, Niš, Serbia; 4 College of Health Science, Academy for Applied Studies Belgrade, Belgrade, Serbia

**Keywords:** Dementia, cognitive impairment, magnetic resonance imaging (MRI), arterial spin labeling

## Abstract

Dementia is a syndrome characterized by multidomain acquired chronic cognitive impairment that has a profound impact on daily life. Neurogenerative diseases such as Alzheimer’s disease or nondegenerative diseases such as vascular dementia are considered to cause dementia. The need for further diagnostic improvement originates from the prevalence of these conditions, especially in developed countries with a predominance of the elderly population. Today, the diagnosis and follow-up of all neurodegenerative diseases cannot be performed without radiological imaging, primarily magnetic resonance imaging (MRI). The introduction of 3T MRI and its modern techniques, such as arterial spin labeling, has enabled better visualization of morphologic changes in dementia. For better diagnosis and follow-up in patients with dementia, various semiquantitative scales have been designed to improve the accuracy of assessment and decrease interobserver variability. Moreover, there is a growing need for MRI in the assessment of novel therapies and their side effects. To better apply MRI findings in the diagnosis of both already developed dementia and its early stages, the aim of this paper is to review the available literature and summarize the specific MRI changes.

## Introduction

Dementia represents a syndrome characterized by multidomain acquired chronic cognitive impairment that has a profound impact on activities of daily living (ADL) [1]. Approximately 35.6 million people worldwide live with dementia, and the number is expected to triple by 2050 [2]. As life expectancy increases, the prevalence of dementia and the economic and social burden on healthcare systems is increasing worldwide [3]. Dementia is only the final stage of the disease, preceded by mild cognitive impairment (MCI). MCI is a stage of subtle cognitive changes that do not impact ADL and represents an important area of interest for drug development researchers [4]. People with MCI can still function independently, whereas the therapy that slows or stops its progression can improve ADL [5, 6]. Given the dementia consequences, there is an urgent need to treat, slow, or halt its progression through the development of improved diagnostic tools and disease-modifying drugs. Magnetic resonance imaging (MRI) has been indispensable in dementia follow-up, diagnosis, and treatment assessment, especially since the introduction of 3T MRI and its modern techniques. MRI may play a role in patient selection for new amyloid-reducing therapies such as aducanumab and also in diagnosing amyloid-related imaging abnormalities (ARIA) associated with amyloid modifying therapies [7]. Accordingly, the purpose of this work is to review the existing literature and describe distinct MRI changes in order to better utilize its results in the diagnosis and prognosis of already-developed dementia and its earliest stages.

## Etiology

Based on the main pathophysiological mechanism, dementia can be classified into neurodegenerative (or proteinopathies) and nondegenerative dementia with numerous entities (Table 1) [1, 8]. Nondegenerative dementia is caused by structural brain pathologies or systemic diseases. The most common nondegenerative dementia is vascular dementia (VaD) [1]. By identifying causes other than degeneration, specific treatments can be initiated, and, in some cases, the cognitive decline can be stabilized or reversed. Neurodegenerative dementias represent the dominant group. Alzheimer’s disease (AD) as the leading phenotype occurs in 60%–80% of late-onset dementias, with a typical presentation that includes progressive memory loss. Another type is Lewy body dementia (LBD), which includes parkinsonism, fluctuating cognition, rapid eye movement sleep behavior disorder, and visual hallucinations. Substantial visuospatial, executive, and attention disorders in the absence of major memory deficits distinguish LBD from other types [1, 5, 9]. Next most common form is frontotemporal dementia (FTD), including three types: the behavioral variant FTD (bvFTD), the nonfluent variant primary progressive aphasia, (nfvPPA), and the semantic variant primary progressive aphasia (svPPA) [1, 9, 10]. In early-onset dementia, under the age of 65, the frequency of occurrence is variable, with AD still predominant, but, with a higher proportion of FTD and LBD. In the elderly, mixed pathology, most commonly consisting of AD and VaD, accounts for up to 30%–40% of all cases [9].

**Table 1:** Neurodegenerative and nondegenerative causes of dementia and entity examples

**Neurodegenerative causes**
Alzheimer’s disease;	Parkinson’s disease;
Frontotemporal dementia;	Posterior cortical atrophy;
Lewy body dementa;	Amyotrophic lateral sclerosis;
Progressive supranuclear palsy;	Multiple system atrophy;
Corticobasal degeneration;	Huntington’s disease
**Nondegenerative** **causes**	
Structural brain lesions	Haemorrhagic and ischemic stroke; primary and secondary tumors; traumatic brain injury; diffuse axonal injury; normal pressure hydrocephalus
Infectious	Meningitis; encephalitis; acquired immunodeficiency syndrome; abscess; syphilis; Creutzfeldt–Jacob disease; Whipple’s disease
Demyelination	Multiple sclerosis
Metabolic	Diabetes mellitus; mitochondrial diseases; lysosomal diseases; obesity
Epilepsy	Therapy resistant; status epilepticus
Systemic	Electrolyte imbalance; hepatic and uremic encephalopathy; vasculitis; connective tissue diseases
Endocrine	Hyperthyroidism; hypothyroidism; Hashimoto’s encephalitis
Deficiency	Vitamin (e.g., Korsakoff’s syndrome) and mineral deficiencies
Intoxication	Alcohol; psychoactive substances; medication-induced (anticholinergic drugs); heavy metals, organic solvents; carbon monoxide
Psychiatric	Schizophrenia; depression; psychosis
Post-anoxia	Choking or strangulation; drowning; chronic smoke inhalation

## Pathophysiology

Neurodegenerative dementias are characterized by a slowly progressive course and the appearance of brain deposits of misfolded proteins [5]. This type of dementia could be caused by more than one abnormally folded protein, and each proteinopathy might have a unique clinical phenotype [11]. These proteins are considered to be the result of an imbalance in production and elimination. They activate brain immune cells, referred to as microglia, which initiate a “defence and repair” mechanism that ensures they are deactivated once their task is complete. This acute inflammatory process can, in an unknown way, progress to chronic inflammation if misfolded proteins accumulate [12]. Advances in the detection of molecular pathology have shown a crossover between phenotypes. There are tau aggregates in AD, but it is considered a secondary tauopathy because β-amyloid (Aβ) is more prevalent [11, 13].

Certain risk factors are included in this pathophysiological process, some of which are modifiable while others are not. The main nonmodifiable risk factors for dementia are older age and genetics [5]. The most commonly described genetic risks for AD include single nucleotide polymorphisms in the apolipoprotein E (*APOE*) region, with carriers of two copies of the *APOE-ɛ4* alleles at increased risk compared with noncarriers [14, 15]. Another genetic risk involves some rare variants of the triggering receptor expressed on the myeloid cells gene (*TREM*), whose carriers have an increased risk for AD. Depending on the present *TREM* variant, microglia may play a protective or deleterious role. Rare variants, such as R47H and R62H, enable microglia deactivation [12]. Examples of modifiable risk factors include lack of physical activity, smoking, low education, social and mental inactivity, high blood pressure, diabetes, and inappropriate diet [3, 16].

## Diagnosis

Dementia remains mainly a clinical diagnosis, with laboratory diagnostics and neuroimaging providing only supportive evidence. Patient assessment begins with a medical history [1]. According to the 2011 NIA-AA revised core clinical criteria and Diagnostic and Manual of Mental Disorders V, dementia can be diagnosed if behavioral or cognitive symptoms: 1) interfere with ADL; 2) represent a decline from prior ADL; and 3) are not caused by a mental illness. Cognitive or behavioral deficits involve at least one domain (memory, executive function, language, visuospatial abilities, and personality/behavior) [6, 17]. The examination should include a detailed mental state analysis. A combination of history-taking and objective cognitive testing is used to identify cognitive impairment. There is a wide range of tests, such as the Mini-Mental State Examination (MMSE) and Montreal Cognitive Assessment (MoCA). Neuropsychological testing represents a gold standard in the assessment of cognitive impairment [1, 17]. The presence and severity of ADL decline are established by conducting a structured or unstructured functional evaluation survey [18].

**Table 2:** MRI dementia protocol

Sequences*	**Purpose**
3D Ax T1W Bravo Cor T1W high resolution	T1W sequences are used to assess the pattern and extent of brain atrophy. Atrophy patterns are best evaluated on the coronal plane, whereas the precuneus is best evaluated on sagittal images. Also, the coronal plane is used for the morphological evaluation of the hippocampus [9, 13].
Ax T2W Prop Cor T2W FRFSE High resolution	T2W sequences are used to identify grey and white matter signal abnormalities, including changes in hippocampal signals, and to determine the degree of vascular damage (lacunae and postischemic parenchymal defects). The T2W coronal sequence should be reported perpendicular to the long axis of the hippocampus to assess the medial temporal lobe [1, 16, 21].
3D FLAIR	3D FLAIR is used to identify oedema, encephalomyelitis, and white matter changes [9].
3D SWAN/SWI**	As many neurodegenerative disorders are associated with accelerated iron deposition and/or microhaemorrhages, SWAN can be applied to detect these diagnostic clues. This sequence is essential in neuroimaging, especially for haemorrhagic lesions, multiple sclerosis, stroke, cerebral amyloid angiopathy, venous anomalies, traumatic injuries, and neoplasms [95].
DWI TENSOR	DWI is an MRI technique that measures the random Brownian motion of water molecules. It is used to show areas with diffusion limitation that indicate acute ischemia, tumor, inflammation, alcoholic encephalopathy, or signaling changes in Creutzfeldt–Jacob disease [9].
3D ASL	ASL measures cerebral blood flow, which uses arterial blood water as an endogenous tracer. Since regional metabolism and perfusion are coupled, ASL may be able to detect functional deficiencies in brain tissue [87].
Gadolinium-based MRI	Gadolinium-based MRI contrast agents may be useful to show whether there is an adequate blood supply to the brain (stenosis, malformation) and in atypical cases, such as suspected infection, tumor, or vasculitis [7, 19].

*The terms of the listed sequences are characteristic of General Electronics Healthcare manufacture. **Susceptibility-weighted imaging (SWI) was initially commercialized by Siemens MRI. To provide an equivalent technique, General Electronic modified T2* and named it SWAN. MRI: Magnetic resonance imaging; 3D Ax T1W Bravo: Three-dimensional axial T1-weighted Brain volume imaging; Cor T1W: Coronal T1-weighted imaging; Ax T2W Prop: Axial T2-weighted propeller sequence; Cor T2W FRFSE: Coronal T2-weighted Fast recovery Fast spin echo; 3D FLAIR: Three dimensional T2-weighted fluid-attenuated inversion-recovery imaging; 3D SWAN: Three-dimensional susceptibility-weighted angiography; DWI: Diffusion-weighted imaging; 3D ASL: Three-dimensional arterial spin labeling.

Additional examinations (biochemistry and vitamins and hormone levels) should be performed to rule out systemic processes. The following tests should be performed in selected patients based on clinical indication and affordability: immunologic and microbiologic blood tests, cerebrospinal fluid (CSF) tests such as Aβ 1 to 40, Aβ 1 to 42, total tau and phospho-tau, electroencephalogram (EEG), and genetic analysis [1, 15, 16].

Neuroimaging includes structural and functional imaging: MRI, computed tomography (CT), and cerebral positron emission tomography (PET) scanning. MRI is preferred over CT due to higher sensitivity for vascular lesions and various forms of dementia. CT may be used for ruling out secondary causes of dementia in the initial diagnosis and also in cases when MRI is contraindicated, or if no previously obtained MRI scans are available for comparison with the present findings [1]. PET diagnostics is also important in the evaluation of patients with cognitive impairment. 18F FDG fluorodeoxyglucose-PET detects hypometabolism patterns in different types of dementia. Amyloid and tau PET with radiotracers that have high affinity for Aβ and tau proteins are less frequently used due to limited availability [1, 18].

## MRI dementia protocol

A 3T field strength scanner should be preferred over 1.5T, whereas a single field strength should be utilized for all patients involved in research to allow for comparability [16]. An advantage of 3T is increased image resolution. The specific MRI receive head coil used should also be taken into account. There is a significant difference between a low and higher number of channels, depending on the manufacturer’s recommendation. For example, Siemens recommends 64 channel head coil for anatomical studies [19]. Researchers should be aware that different head coils can alter the results and thus lead to misinterpretation while comparing the findings [20]. According to a European-wide survey, over 90% of 193 institutions perform MRI dementia protocol [1, 16, 21] which is in agreement with the protocol used at our center (Table 2).

### MRI findings

Understanding the anatomy and physiology of each brain region is required for accurate MRI assessment. The distribution of affected areas in different entities explains the variation in symptoms and imaging patterns [1]. We will attempt to describe the changes commonly found in patients with cognitive decline. It should be emphasized that a different combination of alterations can be found in different types of dementia. However, we will describe these changes in general, whereas pattern changes in different types of dementia will not be included in this review.

### Cerebral atrophy

Atrophy can be represented by detectable changes in early and even presymptomatic stages. In many guidelines, atrophy patterns are included as the core features. Substantial evidence supports the use of those patterns in diagnosis of dementia [22]. 

Medial temporal lobe atrophy (MTA) is a major indicator of several types of dementia and is linked to cognitive decline [23]. It consists of cognition-relevant subregions such as the hippocampus (HC) and structures along the parahippocampal gyrus [24]. In AD, MTA is normally symmetric. However, it can also be asymmetric, as commonly seen in FTD. The entorhinal cortex and the HC are affected in the initial stages. That makes the HC a potentially important prodromal marker as its volume analysis could contribute to early diagnosis. Also, different HC atrophy patterns could potentially aid in the differential diagnosis of different forms of mild dementia, such as LBD, FTD, and Creutzfeldt–Jakob disease (CJD) [25]. However, the precision for distinguishing AD from other types of dementia is lower than from controls, e.g., less severe atrophy is more prevalent in LBD [9].

Frontal lobe atrophy has been mostly connected to FTD, a condition that is clinically distinguished by changes in behavior or language. Atrophy first occurs in the anterior cingulate, insula, and frontal lobes [26]. Bilateral and possibly asymmetrical frontal and temporal atrophy with an anterior-to-posterior gradient is typical. Simultaneously atrophying caudate head may cause a widening of the frontal horns that is disproportionate. Orbitofrontal sulci broadening may be considered an early sign [9]. Although there may be variations, atrophy typically correlates to clinical subtypes. For example, nvPPA is focused in left perisylvian parts, including the inferior, opercular, and insular areas, whereas svPPA is localized predominantly in ventral and lateral parts of the anterior temporal lobes, along with the anterior HC and the amygdala. Both subtypes are characterized by a left-hemispheric dominance [1].

Posterior/parietal atrophy (PA) is characterized by early and substantial visual and visuospatial impairment, as well as less apparent memory loss, and is linked with atrophy in the parieto-occipital and posterior temporal regions. Typically, the atrophy is asymmetric, with the right side being more affected. The PA most commonly occurs in atypical AD and logopenic variant PPA. In LBD, the PA is not as common as in AD, even though occipital hypometabolism is a characteristic LBD pattern on PET [27, 28]. Corticobasal degeneration, subcortical gliosis, and prion disease are entities less frequently associated with PA [9, 28]. In individuals with AD, Lehmann et al. [29] found substantial PA in the absence of visible atrophy in the medial temporal lobe (MTL), especially in younger individuals. Since MTA is considered an AD diagnostic sign, this may imply that some patients may not be diagnosed if only MTA was evaluated.

Deep gray matter (GM) atrophy is also an important imaging indicator. As in the case of the cortex, the involvement of the deep GM also displays various patterns. The caudate nucleus and the thalamus are commonly affected in bvFTD [18]. In LBD, the thalamus is also affected, but predominantly in the ventral–dorsal and pulvinar regions [30]. In AD, thalamic involvement includes asymmetric atrophy in the ventrolateral and ventromedial nuclei correlating with the severity of the disease. Also, the asymmetric atrophy of the caudate nucleus can be found in AD patients [18, 31]. Although some deep GM atrophy patterns have been established, there are still some discrepancies between studies; hence, further research is needed. Another important finding includes iron deposition in basal ganglia (BG). Some data show that verbal memory and executive function can be affected by iron accumulation. Even though iron depositions are commonly considered a Parkinson’s disease feature, if detected, it should be noted in radiological reports as another possible cause of dementia [32].

### Microbleeds

**Figure 1.**
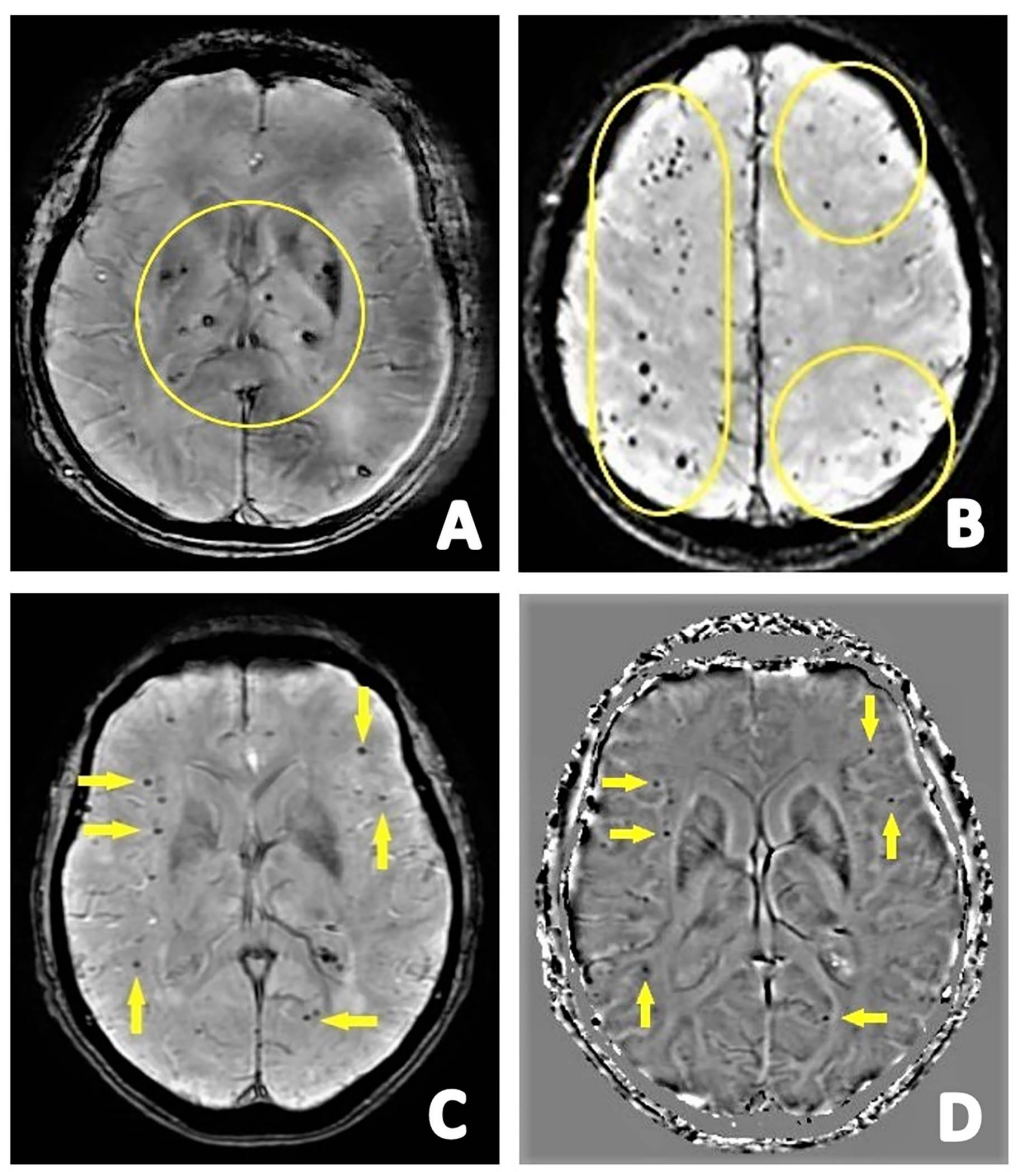
The first row presents axial SWAN images showing: (A) multiple CMBs in the deep GM (circle) indicating probable hypertensive disease; (B) multiple lobar CMBs (circle) indicating probable CAA. The second row shows: (C) axial SWAN images with multiple lobar CMBs (arrows) corresponding with (D) filtered phase images for confirming CMBs (arrows). SWAN: Susceptibility-weighted angiography; CMBs: Cerebral microbleeds; GM: Gray matter; CAA: Cerebral amyloid angiopathy.

Cerebral microbleeds (CMBs) represent persistent deposits of blood breakdown products, especially hemosiderin. On T2, they are detected as focused spots of signal loss, less than 10 mm in diameter, that grow in size on T2*-weighted gradient echo images (“blooming” effect) (Figure 1) [33, 34]. An alternative, more sensitive sequence is susceptibility-weighted angiography (SWAN), with greater “blooming” with more apparent but possibly unevenly formed CMBs. Filtered phase SWAN is used to distinguish CMBs from calcifications. On SWAN, both of these can be seen as hypointense. On the other hand, in the filtered phase, calcifications (diamagnetic) can be seen as hyperintense, whereas CMBs (paramagnetic) as hypointense lesions (Figure 1). However, depending on whether the filtered phase SWAN is used in a right- or left-handed coordinate system, CMBs may appear hyperintense on a phase image [35]. They have been associated with several pathologies, such as cerebral small vessel disease (cSVD), stroke, brain injury, radiation, and VaD. Although CMBs might be asymptomatic, early detection is critical in risk assessment of late cerebrovascular illness and cognitive deterioration [36].

The classification of CMBs includes three categories: deep (BG, thalamus, internal and external capsule, and corpus callosum) and periventricular white matter (WM), lobar (frontal, temporal, parietal, and occipital), and infratentorial (brainstem and cerebellum). Localization of CMBs contributes to the differential diagnosis between cerebral amyloid angiopathy (CAA) and hypertensive disease [35]. Lobar localization is more common in CAA diagnosis, where a preferential parietooccipital distribution has been reported. The deep category is more affected by the hypertensive disease of the small penetrating arteries [35, 37]. A solitary CMB in any brain region cannot be regarded as clinically significant. However, several lobar CMBs in a person over 55 years of age without additional explanations indicate a probable CAA. Multiple lobar CMBs are frequently detected in patients who meet the AD clinical criteria due to common CAA and AD co-occurrence. If superficial siderosis is also present, the diagnosis of CAA is strengthened. Cerebellar CMBs can occur in both CAA and hypertension and thus do not significantly contribute to diagnosis [9].

### White matter hyperintensities

WM hyperintensities (WMHs) are visualized as zones of a high-intensity signal on T2W and FLAIR sequences. They represent a key manifestation of cSVD and are widely detected among the elderly [34]. In their meta-analysis, Hu et al. [38] showed that WMHs are related to an elevated risk of cognitive impairment and might become an indication for imaging in dementia. However, there is a clinical and radiological disparity between whole-brain WMHs and cognitive impairment, as some people with severe WMHs can still function well [39].

In previous histopathological studies, WMHs corresponded to several underlying lesions. Smooth, periventricular WMHs (PWMHs) are associated with moderate, nonischemic changes, whereas more severe lesions are associated with irregular and confluent WMHs. On the other hand, confluent and deep WMHs (DWMHs) are related to cognitive impairment, mortality, and an increased risk of stroke, whereas PWMHs are not [40]. Researchers have attempted to separate WMHs into distinct lobes, but the findings were inconclusive [9, 39, 41]. According to a retrospective study, frontal WMHs are observed more frequently in AD patients than in the elderly. Similar as in AD, WMHs on T2 are also seen more frequently in LBD than in controls [9]. Another study found that higher DWMHs loads in frontal regions are related to lower memory and executive performance in cognitively unimpaired persons predisposed to AD risk factors [41]. Cross-sectional research also showed that WMHs near the frontal horns mostly affect executive function, parieto-temporal WMHs near the posterior horns affect memory, whereas WMHs in the upper deep WM, including the corticospinal tract, affect motor speed performance [39].

### Lacunes and dilated perivascular spaces

A lacune is a lesion 2–20 mm in diameter, typically affecting the subcortical WM and the deep GM. It is a fluid-filled gliotic cavity that occurs prior to infarcts of the deep perforating arteries. Lacunes appear with CSF signal intensity on all sequences, bordered by a T2 hyperintensity rim. They should not be confused with dilated perivascular spaces (PVS), i.e., spaces filled with CSF that surround perforating arterioles and venules as they pass through the parenchyma out from the subarachnoid space. Dilated PVS is typically formed by volume loss in the surrounding tissue, with a predisposition for the BG [9, 42, 43]. Both are considered cSVD manifestations. Areas smaller than 2 mm, presented in the lower third of the corpus striatum and showing intensified CSF, can help in differentiating dilated PVS from lacunes [34]. There is further indication that lacunes may be linked to cognitive decline, death, and poor clinical outcomes. Lacunes were independently associated with cognitive function and strongly predictive of rapid global functional decline in a sample of older persons living independently [40].

**Figure 2.**
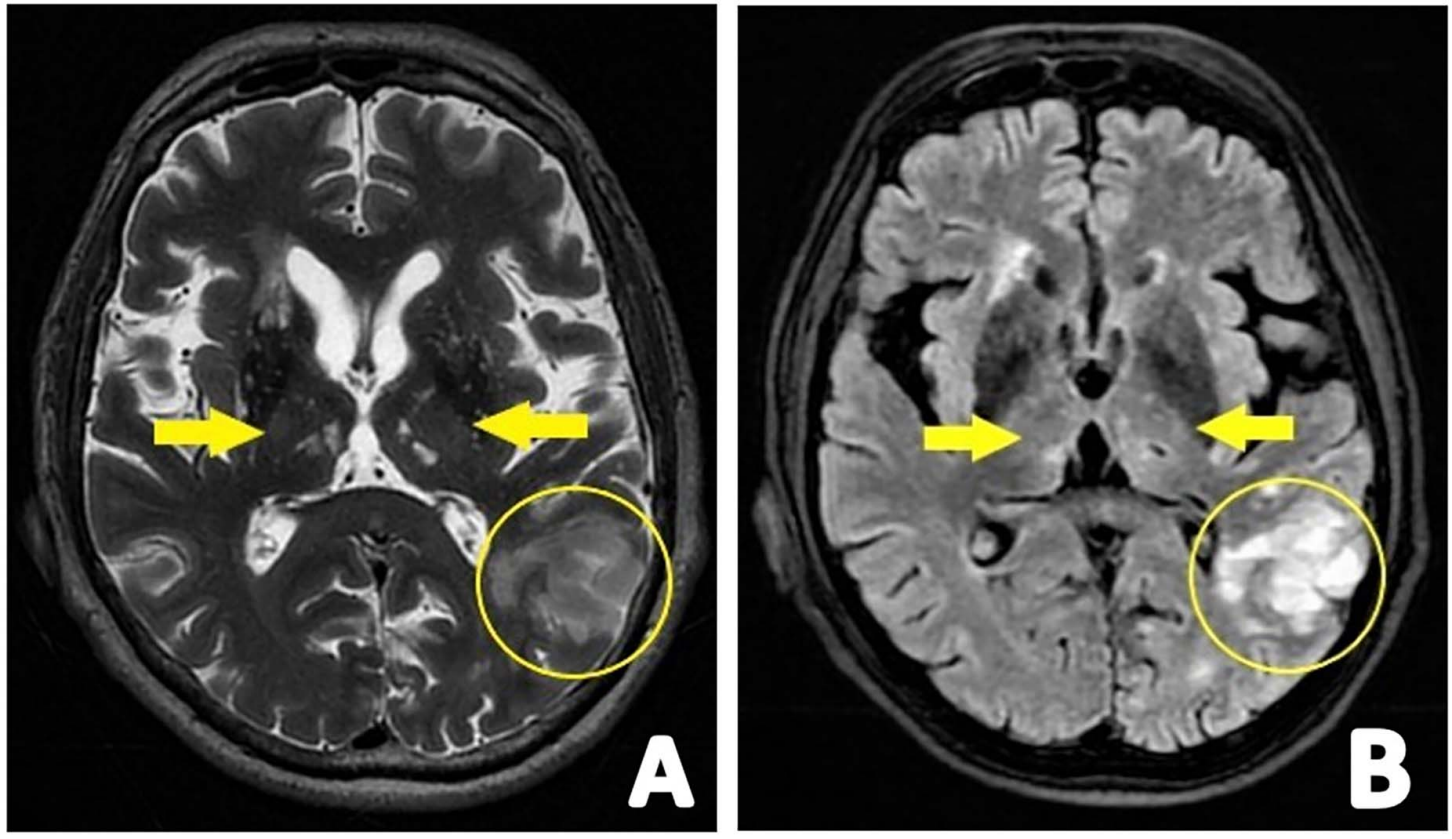
**(A) Axial T2W and (B) axial FLAIR images showing strategic infarctions in both thalami and in the parieto-occipital watershed area.** Images were obtained in a male patient, age 71, with clinical manifestation of vascular dementia. FLAIR: Three-dimensional T2-weighted fluid-attenuated inversion-recovery imaging.

### Strategic infarcts

We have previously described the effects of lacunar infarcts on cognition. However, another location-dependent type of infarction can also be found in patients with dementia, especially VaD (Figure 2). Acute cognitive deterioration may be presented with a large cortico-subcortical ischemic lesion. However, dementia progression due to a stroke may be driven by infarcts in “strategic” regions that are important for normal cognitive functioning. Research on the relationship between brain lesion location and cognition found that the degree of cognitive impairment following stroke may be dependent on its features, such as type, size, number, location, and severity [9, 44]. Interestingly, a multivariate lesion-symptom mapping study showed that strategic infarction correlated with compromised cognition was of smaller size [45]. Strategic infarct localization includes the angular gyrus, bilateral anterior cerebral artery, paramedian thalamic, inferior MTL, parieto-temporal and temporo-occipital association areas, and angular gyrus, superior frontal, and parietal watershed areas in the dominant hemisphere. Also, HC or bilateral/unilateral thalamic infarctions have been found to cause dementia [9, 44]. In the previously mentioned mapping study, the angular gyrus, BG, and the WM surrounding BG, predominantly on the left side, had the highest correlation with global decreased cognition [45].

**Figure 3.**
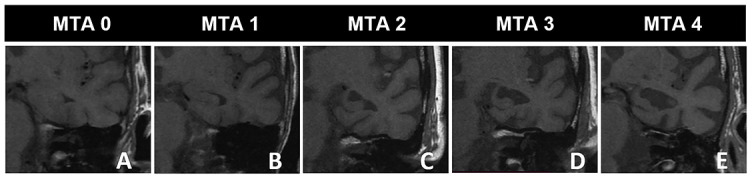
** Coronal T1W images show different degrees of medial temporal lobe atrophy in 5 different patients, aged 64–87 years, with Alzheimer’s disease clinical presentation.** Using the Scheltens scale, the medial temporal lobe is assessed on coronal planes: (A) MTA0 – normal width of the choroid fissure, the temporal horn, and a normal HC height; (B) MTA1 – mild widened choroid fissure, normal temporal horn, and HC height; (C) MTA2 – moderately widened choroid fissure, mild temporal horn enlargement, and a mild reduction in HC height; (D) MTA3 – markedly widened choroid fissure, a moderate enlargement of the temporal horn, and a moderate reduction in HC height; (D) MTA 4 – markedly widened choroid fissure, enlargement of the temporal horn, and a reduction in HC height. MTA: Medial temporal lobe atrophy; HC: Hippocampus.

## Standardized assessment

As qualitative evaluation could result in increased ambiguity and interobserver variability, several efficient, repeatable, and cost-effective semiquantitative scales have been proposed [13]. They have been created to provide reproducible semiquantitative volume loss evaluation. The scales can be useful in everyday imaging and may increase diagnostic precision [46]. A variety of scales have been developed, not only for atrophy assessment but also for CMBs and WMHs. We will mention only a few that are the most common in the literature. It is also important to include advanced techniques, such as arterial spin labeling (ASL), which has become increasingly important in the evaluation of dementia and volumetry analysis.

A radiological report should contain [46]:

- Scales, at least: MTA scale; Global cortical atrophy (GCA) scale; Koedam score; Fazekas scale;
- Extent and location of infarcts;
- CMBs;
- WMHs;
- Other alterations (tumor, hydrocephalus, subdural hematoma, and other possible causes);
- Comparison with prior assessments.

**Table 3:** Scheltens scale for medial temporal lobe assessment

**MTA**	**Characteristics**
0	Normal choroid fissure width, temporal horn width, and the HC volume
1	The choroidal fissure mildly widened
2	Moderately widened choroid fissure, minor temporal horn expansion, and modest HC volume loss
3	Considerably expanded choroid fissure, moderate temporal horn expansion, and moderate HC volume loss
4	A significantly expanded choroid fissure, a significantly enlarged temporal horn, and a significantly reduced HC volume

HC: Hippocampus; MTA: Medial temporal lobe atrophy.

### MTA scale

The degree of MTA can be assessed using the Scheltens scale (Table 3) on coronal planes perpendicular to the long axis of the HC [42]. Figure 3 shows the Scheltens scale, which focuses on three MTL features: the choroid fissure, the temporal horn width, and the HC height. Both sides are evaluated independently [47, 48]. Over time, the Scheltens scale has been modified. For example, Galton et al. [49] expanded it to include non-HC structures. The scale has been divided into two sections, the first utilizing the Scheltens scale and the second designed to grade the anterior, non-hippocampal medial (the parahippocampal gyrus), and lateral temporal regions of each hemisphere independently [47, 49]. Urs et al. [47, 50] and Duara et al. [47, 51] have independently created a grading system focused on a single identifiable landmark slice at the level of the mamillary bodies.

**Figure 4.**
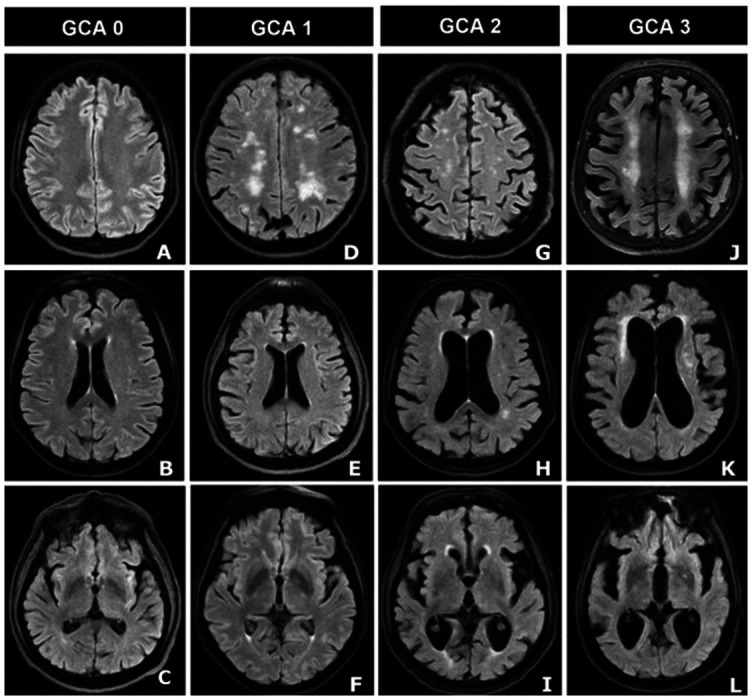
**Axial FLAIR images of 4 different male patients, aged 66, 72, 86, and 82 years, with clinical Alzheimers’s disease manifestation showing global cortical atrophy and ventricular dilatation in different stages.** Using the Pasquier scale with O’Donovan addition in the assessment of ventricular enlargement, the first column shows: (A) normal volume of the gyri and width of the sulci, (B) normal dilatation of lateral ventricles, and (C) normal dilatation of the third ventricle. The second column shows: (D) mild atrophy with a still normal volume of the gyri but some open sulci, (E) mild dilatation of lateral ventricles, (F) mild dilatation of the third ventricle. The third column shows: (G) moderate brain atrophy with a reduction of gyri volume, and enlargement of the sulci, (H) moderate dilatation of lateral ventricles, (I) moderate dilatation of the third ventricle. The fourth column shows: (J) severe atrophy with severely reduced gyri, and enlarged sulci, (K) severe dilatation of lateral ventricles, (L) severe dilatation of the third ventricle. FLAIR: Three-dimensional T2-weighted fluid-attenuated inversion-recovery imaging.

### GCA scale

The Pasquier scale, previously known as the GCA scale [47, 52], assesses atrophy in 13 distinct brain areas (frontal, parieto-occipital, and temporal) and assigns a score (0–3) to each region (Table 4). GCA is best evaluated on axial FLAIR images (Figure 4) [42, 53]. At first, Pasquier et al. [52], described a rating scale where only gyral volume and sulcal dilatation were assessed. Later, O’Donovan et al. [54]. added a scale to assess ventricular enlargement as a marker of GCA on the Pasquier scale. Each hemisphere was scored independently, and the results were added together to get an overall value ranging from 0 to 39 [47, 54]. The ventricles evaluation has great reliability; nevertheless, the scale is less effective in terms of differential diagnosis, possibly due to a significant range of ventricular size among healthy persons [47]. This scale is more likely to be confounded by age compared to other scales. However, its diagnostic effectiveness can be enhanced by the application of age-specific cutoffs [53, 55].

**Table 4:** Pasquier scale for global cortical atrophy assessment

**GCA**	**Characteristics**
0	Normal volume of the gyri, sulci width, and ventricle dilatation
1	Mild atrophy with still normal gyri volume, however, with some slightly open sulci and mild ventricular dilatation
2	Moderate brain atrophy with reduced gyri volume, increased sulci, and moderate ventricular dilatation
3	Severe atrophy with significantly shrunken gyri, enlarged sulci, and dilated ventricles

GCA: Global cortical atrophy.

**Table 5:** Koedam score for posterior atrophy assessment

**Koedam**	**Characteristics**
0	The posterior cingulate is closed, as also are the parieto-occipital sulcus, the parietal lobe sulci, and the precuneus
1	Mild posterior cingulate and parieto-occipital sulcus widening, with mild parietal lobe and precuneus atrophy
2	Significant expansion of the posterior cingulate and parieto-occipital sulcus, as well as significant atrophy of the parietal lobes and precuneus
3	End-stage atrophy with evident sulci expanding and knife-blade atrophy of the parietal lobes and precuneus

### Koedam score

**Figure 5.**
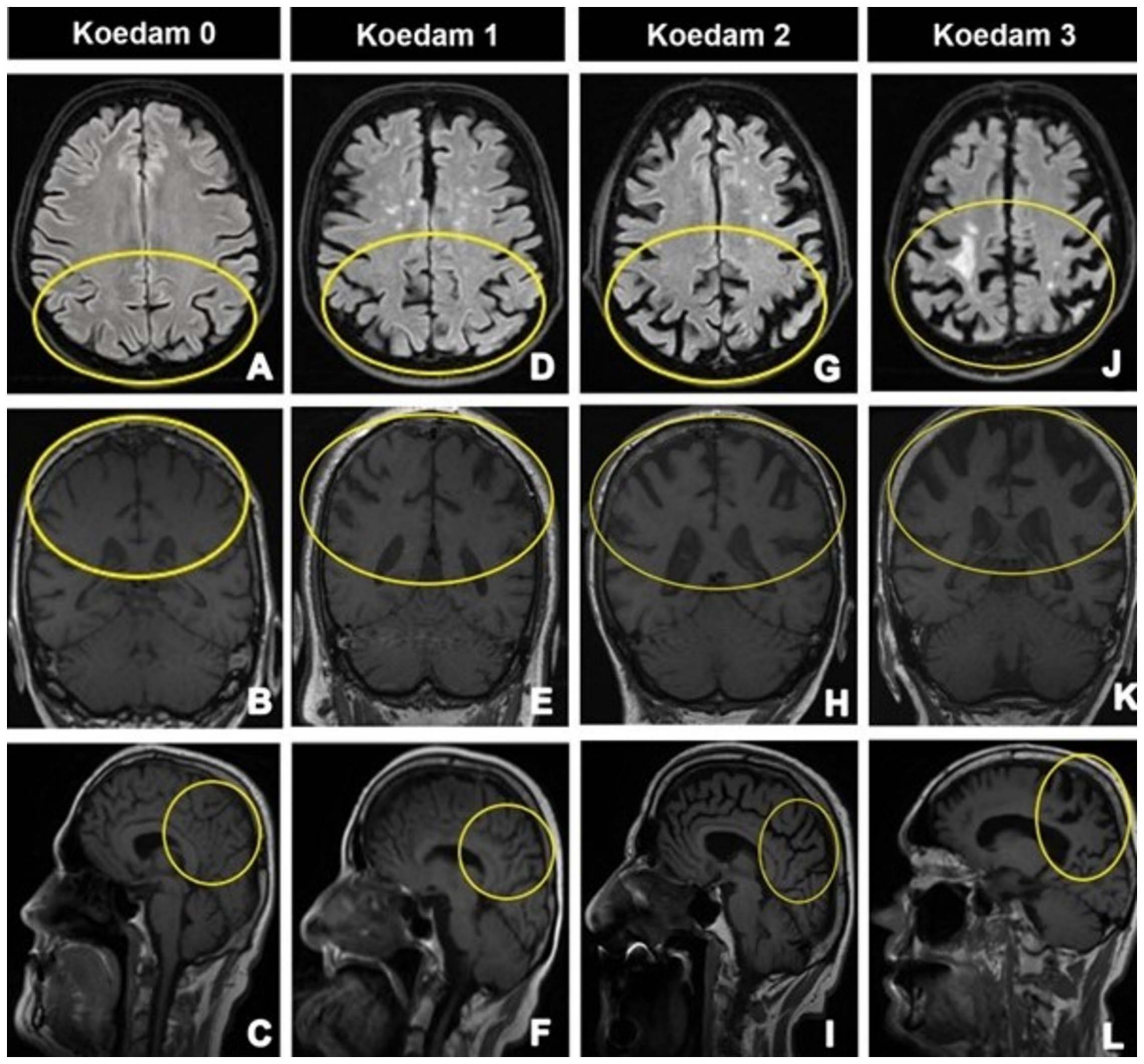
**Axial FLAIR, coronal T1W, and sagittal T1W images show parietal atrophy scale. Images are of 4 different patients with Alzheimer’s disease, aged 70, 87, 86, and 83 years.** First column: Koedam 0: (A)–(C) show the closed posterior cingulate, parieto-occipital, and the parietal lobe sulci, and the normal precuneus (yellow circles). Second column: Koedam 1: (D)–(F) show mild posterior cingulate, parieto-occipital, and the parietal lobe sulcal widening, and the mild atrophy of precuneus (yellow circles). Third column: Koedam 2: (G)–(I) show substantial posterior cingulate, parieto-occipital, and the parietal lobe sulcal widening, and the substantial atrophy of precuneus (yellow circles). Fourth column: Koedam 3: (J)–(L) show extremal posterior cingulate, parieto-occipital, and the parietal lobe sulcal widening, and the mild atrophy of precuneus, knife-blade precuneus atrophy (yellow circles). FLAIR: Three-dimensional T2-weighted fluid-attenuated inversion-recovery imaging.

Koedam et al. [56]. proposed a visual rating scale for PA evaluation. The Koedam scale focuses on the posterior cingulate and the parieto-occipital sulcus, the precuneus, and the parietal lobe cortex (Table 5). The left and right hemispheres are scored independently, and each imaging plane gets a separate score [47, 56]. Figure 5 depicts the Koedam score example. All landmarks have been rated in different orientations: widening of the posterior cingulate and parieto-occipital sulcus, as well as precuneus atrophy on sagittal, sulcal dilatation in the parietal lobes and enlargement of the posterior cingulate sulcus on axial, and widening of the posterior cingulate sulcus and parietal lobes on the coronal plane [42, 56]. The Koedam score should be utilized as an additional measure in differential diagnosis, particularly in early-onset AD [57]. The sensitivity and specificity in differentiating AD from the control group were 58% and 95%, respectively [56]. In the study by Yuan et al. [62] these results were superior to the results in mild cases and inferior to those in intermediate and severe AD cases.

### Fazekas scale

**Figure 6.**
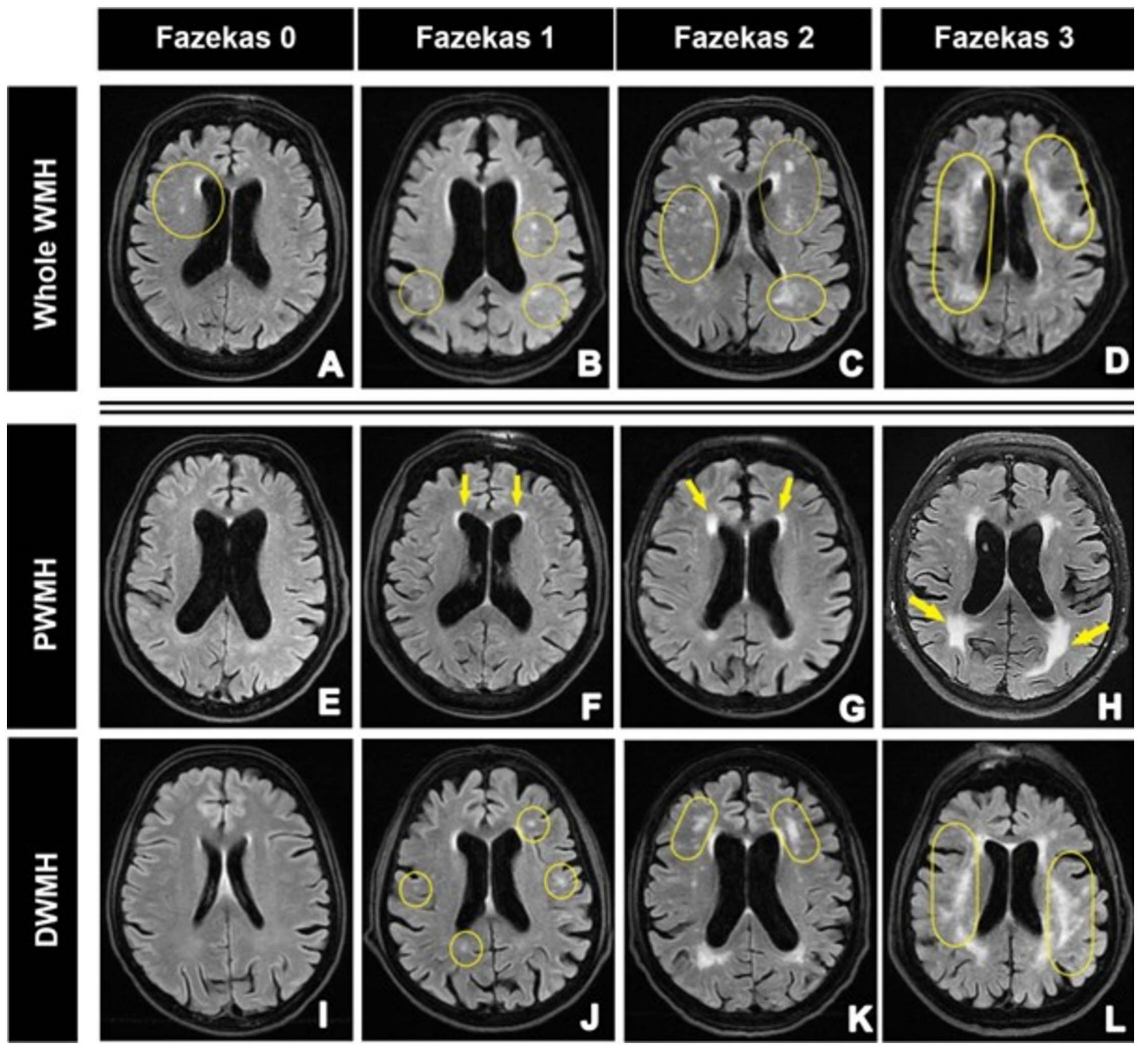
**Axial FLAIR images of different patients, mostly with Alzheimer’s disease and vascular dementia clinical presentations (aged between 55 and 87 years) show Fazekas scales**. The first row: whole WMHs: (A) single WMH (circled yellow); (B) multiple WMHs (circled yellow); (C) WMHs that start to bridge (circled yellow); (D) large confluent WMHs (circled yellow). The second row: PWMH: (E) absent WMH; (F) “cap and bands” (yellow arrows); (G) smooth periventricular hallo (yellow arrows); (H) PWM extends into DWM (yellow arrows). The third row: DWMH: (I) absent WMHS; (J) punctate foci (circled yellow); (K) WMHs that start to confluent (circled yellow); (L) large confluent WMHs (circled yellow). FLAIR: Three-dimensional T2-weighted fluid-attenuated inversion-recovery imaging; WMH: White matter hyperintensities; DWMH: Deep white matter hyperintensities; PWMH: Periventricular white matter hyperintensities.

Fazekas scale assessment is the most often used rating scale for determining the severity of WMHs. It also represents a four-step scale from 0 to 3 (Table 6). The whole WMH assessment can be seen in Figure 6. It plays a role in evaluating post-stroke cognitive impairment since grades 2 and 3 have been associated with disability 90 days and one year after ischemic stroke [9]. Also, The Radboud University Nijmegen Diffusion Tensor and Magnetic Imaging Cohort study demonstrated that a greater grade indicates a greater pathologic burden, corresponding to a lower MMSE score and a decline in cognitive functioning [43].

**Table 6:** Divided Fazekas scale for assessment of the deep and periventricular white matter

**Fazekas**	**0**	**1**	**2**	**3**
Whole WMHs	Sporadic or nonpunctate WMHs	Multiple punctate WMHs	Confluent lesions bridging the punctate WMHs	Extensive confluent WMHs
PWMHs	Absent WMHs	Thin WMHs running along ventricular horns (caps and bands)	More extensive WMHs (smooth hallo)	Irregular periventricular signal extending into deep WM
DWMHs	Absent WMHs	Punctate foci	Foci that started to confluent	Large confluent areas of WMHs

WMHs: White matter hyperintensities; PWMHs: Periventricular white matter hyperintensities; DWMHs: Deep white matter hyperintensities.

There is a division of the Fazekas scale into DWMHs and PWMHs scales (Figure 6) [58]. Some authors use this divided form, while others use only the overall Fazekas score for the whole WM. As shown in Table 6, there are some differences between the whole and divided scale. The whole scale considers DWMHs to a greater extent, whereas the first two grades of PWMHs are almost considered normal in the whole WM form of the Fazekas scale. Research has found that DWMHs have greater and more correlative diagnostic value to clinical presentation value (see: White matter hyperintensities); however, specific guidelines on which scale to use have not yet been established, and in both scales, there is a possibility to underdiagnose or overdiagnose patients [59].

### Cutoffs

The first cutoff values for differentiating normal from abnormal atrophy patterns included:

Asymmetrical and focal atrophy on the GCA scale should be regarded as abnormal, as well as a score of 3 in all individuals or a score of 2 in those over the age of 75 [9]; regarding the MTA scale, the initial suggestion included an age-adjusted cutoff. A score of 2 in either of the two hemispheres is considered abnormal below the age of 75, whereas a score of 3 is considered abnormal above the age of 75 [48]; Fazekas et al. [58] recommended a cutoff of 2 as abnormal; Fazekas score of 1 is considered normal, but 2 and 3 suggest the existence of cSVD. A score of 3 is considered abnormal at any age, indicating a high percentage of WM lesions and contributing to the VaD clinical criteria [9].

As these cutoffs have not shown sufficiently high sensitivity and specificity, many authors have attempted to design new ones, taking into account *APOE*, age, and other factors. For example, Claus et al. [60] suggest decade-specific cutoffs (<65, 65–74, 75–84, and ≥85 years); however, after 85 years of age, their utility is reduced. Using age-specific cutoffs, Wei et al. [61] demonstrated high sensitivity and specificity for discriminating AD from controls but not for discriminating MCI from controls. By combining scales, Yuan et al. [62] showed an improvement in sensitivity and specificity of differentiation between AD patients and controls. Since a unique agreement on cutoff values has not yet been established, further research is necessary.

### Volumetry

**Figure 7.**
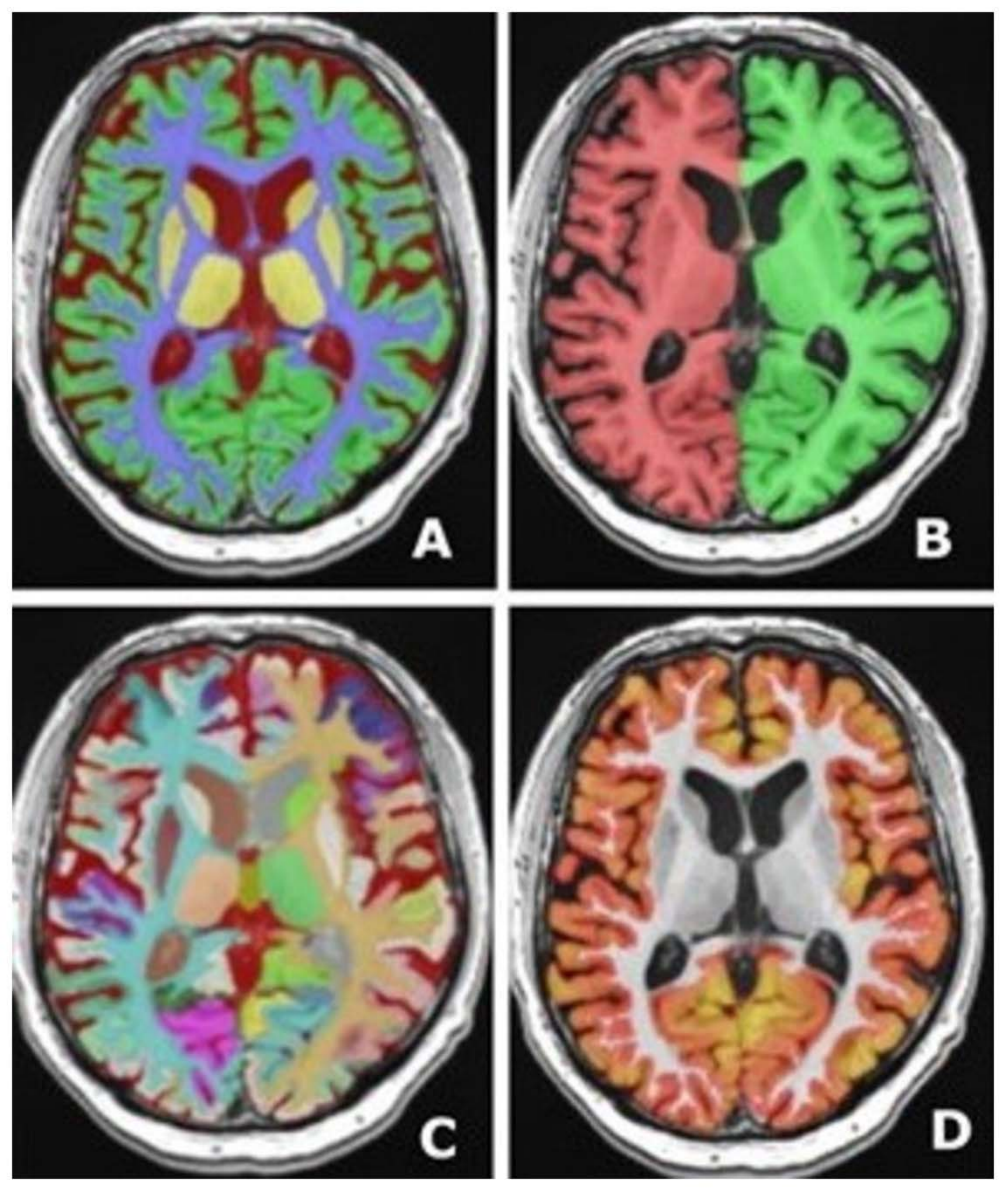
**Volumetric analysis using an automated MRI brain volumetry system.** (A) Tissue segmentation – for volumetric assessment of white and gray matter; (B) Macrostructures – for volumetric assessment of the hemispheres, and their white and gray matter volume; cerebellar volume is also included; (C) Structure segmentation – detailed volume assessment of each gyrus, and basal ganglia; (D) Cortical thickness – for the evaluation of cortical volume in different regions. MRI: Magnetic resonance imaging.

Specialized programs that could provide volumetric evaluation are also available. Volumetry is not only used for evaluating the GM and WM volume but also for CMBs, WMHs, and ischemic lesions [13]. The volBrain is an example of volumetry software, and its segmentation process is presented in Figure 7 [63]. Another commonly used volumetric tool is FreeSurfer [64]. NeuroQuant, the FDA-approved software for routine dementia assessment, is also worth noting. It contains a standardized assessment, with the possibility of comparing MRI scans to national databases with comprehensive patient information [65]. Pemberton et al. [66] found that using quantitative reporting in addition to visual evaluations enhances sensitivity and precision for identifying volume loss compared with visual assessments alone. Progression of MTA, quantified through the HC volume, has been investigated in both control and preclinical, prodromal, and probable AD patients [67]. Volumetry and MTA comparisons have revealed correlations ranging from acceptable to excellent. Hippocampal volumetry can help differentiate HC sclerosis in epilepsy and dementia, especially FTD and AD. Volumetric analysis of HC subfields showed that sclerosis develops in different parts of the HC [68]. Another important differential diagnosis includes some other causes, such as normal pressure hydrocephalus. Volumetry has improved diagnostic accuracy of differentiating normal pressure hydrocephalus from AD and Parkinson’s disease but it could also be used to predict outcomes after shunting [69]. Based on the European-wide survey, methods such as volumetry are currently mostly applied in research settings, and only 5.7% of all centers perform it regularly, even though these programs were introduced years ago [21].

## Normal aging

In older persons, the cognitive decline spectrum ranges from normal age-related cognitive decline to subjective cognitive impairment (cognitive complaint with normal cognitive screening test) to MCI and dementia [17]. Hence, the question is, what specific degree of the abovementioned MRI changes should be considered normal? Brain atrophy and cSVD imaging markers were the most prevalent imaging findings in aging that coincide with those reported in dementia [9]. The basic rule for distinguishing age-related atrophy from neurodegeneration is that age-related atrophy is typically symmetric and widespread, whereas dementia exhibits unique patterns and asymmetries. Semiquantitative visual rating scales could assist in distinguishing between “normal” and “abnormal.” In addition, finding a solitary CMB is not considered relevant; however, multiple ones should arise suspicion [35]. On the contrary, cerebrovascular alterations are prevalent in elderly. Some criteria to diagnose VaD proposed by the National Institute of Neurological Disorders and Stroke and Association Internationale pour la Recherché et l’Enseignement en Neurosciences are: a) confluent WMHs affecting 25% of the total WM; b) lacunar infarcts affecting the frontal WM, multiple BG, and both thalami; or c) large-vessel infarcts affecting strategic regions [9]. Nevertheless, every change should be noted and interpreted along with the clinical findings.

## From mild cognitive impairment to dementia

MCI diagnostic criteria include impaired cognitive performance in one or more domain; however, with proper ADL [17]. The yearly rate of progression to dementia is approximately 15% [70]. But how can we recognize a patient transitioning from MCI to dementia on neuroimaging? According to quantitative MRI studies, HC atrophy is detectable before dementia starts and worsens with conversion to clinically manifest disease. A longitudinal study including MCI patients has shown that memory decline over time was associated with bilateral HC atrophy, while left HC atrophy predicted a visuospatial decline [71]. Pyun et al. [72] reported that PA in amyloid-positive MCI is substantially linked with dementia development, regardless of the presence of MTA. This demonstrates the predictive usefulness of PA for progression in amyloid-positive MCI patients and suggests that PA can be used as a progression predictor from amyloid-positive MCI to dementia.

## Advanced MRI techniques

Diffusion-weighted MRI images (DWI) are applied to identify regions with diffusion limitations. Tissues that are highly cellular or affected by cellular swelling have lower diffusion coefficients than normal. DWI has been particularly significant for the diagnosis of CJD and VaD, and it also allows the detection of differential diagnoses, such as ischemia, encephalitis, and neoplasia [9]. Shiga et al. [73] concluded that DWI has higher sensitivity in detecting CJD than FLAIR, T2W, EEG, CSF proteins, or neuron-specific enolase. DWI provides quantitative lesion assessment by evaluating the apparent diffusion coefficient (ADC). In CJD, DWI shows limited diffusion in at least two cortical areas (“ribboning”) and/or restricted diffusion in the caudate nucleus, followed by the putamen and thalamus. Using DWI would enable distinguishing different types of CJD, such as “pulvinar sign” in variant CJD—a signal on FLAIR and DWI of higher intensity in the posterior than in the anterior putamen [74]. Taoka et al. [75] noted that ADC of the uncinate fascicles also linked well with cognitive performance and appeared to be useful as biomarkers for detecting the progress of AD.

**Figure 8.**
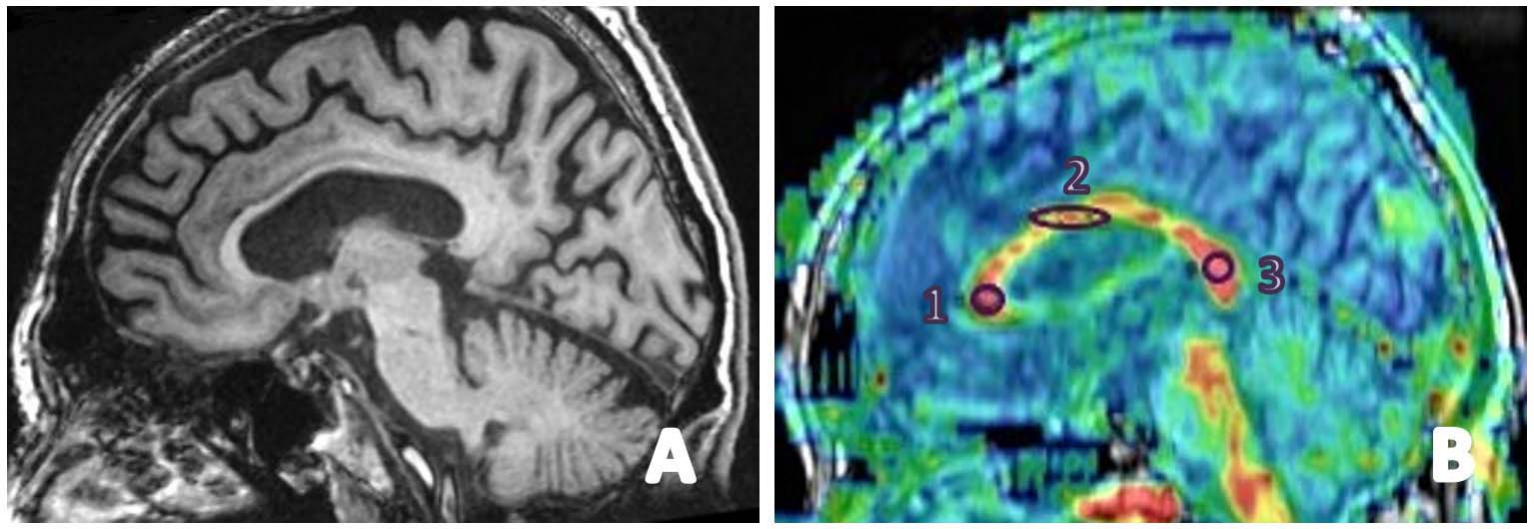
**The figure shows fractional anisotropy of the corpus callosum in a male patient with clinical presentation of Lewy body dementia**. (A) A T1W image in the sagittal plane; (B) a fusion image of T1W and color-coded fractional anisotropy maps made on the General Electronics 3T MRI workstation. Fractional anisotropy (FA) of the corpus callosum with region of interest put on: 1) genu with FA ═ 0.72, 2) corpus with FA ═ 0.50, and 3) splenium with FA ═ 0.85. MRI: Magnetic resonance imaging.

Diffusion tensor imaging (DTI) studies on the microstructural features of WM and its derived metrics have been widely used in the investigation of AD and MCI. Derived metric, such as fractional anisotropy (FA), is indicative of water diffusion along WM tracts that is radially constrained by the myelin sheath enclosing axons (Figure 8) [76].

Different parts of corpus callosum are affected in various types of dementia since different parts of the cortex are affected, and degeneration of WM fibers represents a consequence of neuronal loss in the cortex [77]. Shim et al. [78] demonstrated the genu FA values as an important predictor of global cognition in all dementia and MCI groups. Atrophy and FA alteration in the genu and splenium are commonly seen in AD, and these abnormalities are considered to occur in the initial stage of AD. The genu receives axons from the prefrontal cortex and myelinates later than the splenium, which receives axons from the temporoparietal lobes, which typically exhibit atrophy and hypometabolism in AD.

Derived DTI metrics are also the sensitive cSVD indicators for detecting early cognitive deterioration. Subtle changes in some WM tracts can be identified from the preclinical stage of vascular MCI, with a localized to generalized spreading pattern during development [79].

**Figure 9.**
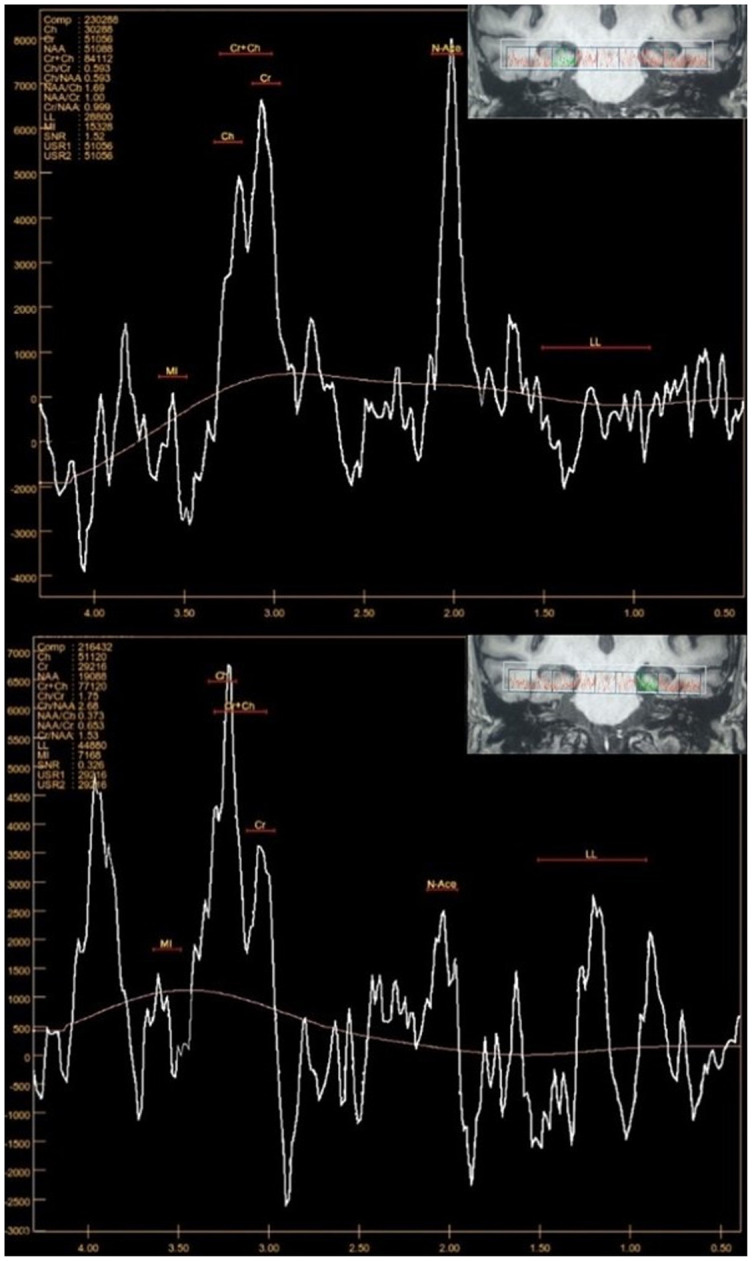
**Multivoxel magnetic resonance spectroscopy, using mean echo time (Te 144 ms), was performed on a female patient with suspected Alzheimer’s disease.** Region of interest has been put manually over the right and left hippocampus. The spectroscopy in the right hippocampus region (upper image) shows the metabolic ratios NAA/Cr – 1.0 and Cho/Cr – 0.59. On the left (lower image) is a decrease in NAA values, with an increase in Cho values with metabolic ratios NAA/Cr – 0.65 and Cho/Cr – 1.75, and an elevation in MI values. NAA: N-acetyl aspartate; MI: Myo-inositol; Cho: Choline; Cr: Creatine.

Magnetic resonance spectroscopy (MRS) provides a noninvasive biochemical examination of the brain in vivo (Figure 9). Metabolites N-acetyl aspartate (NAA), myo-inositol (MI), total choline (Cho), and total creatine (Cr) are present in the brain in amounts suitable for detection and have been commonly studied. It has been shown that in AD, the NAA peak decreases while MI and Cho peak increases, particularly in the posterior cingulate and HC. This pattern is inconsistent across the studies, and only the NAA drop seems to be significant [80]. The MRS results have shown some variation, which is higher in the MTL due to the bone at the base of the skull as well as the air in the sinuses, which is why its clinical efficacy in dementia is lacking [1]. In the literature, limitations to MRS usage are technical variances in data collection, varied voxel composition, the existence of underlying pathology in each voxel, constraints of lower field MRS, and voxel location changes. Even if there are some alternatives (ultra-high field MRI), standardization is needed [81, 82].

Some commercial MRS processing software packages use linear-combination modeling (LCM) of in vitro spectrum to maximize the use of prior information in the analysis. LCModel is among the most quoted. However, the application of software also involves some drawbacks, such as a moderate agreement between algorithms for the same metabolites and poor understanding between standardized model parameters and MRS results [83]. Although MRS does not add much to the initial diagnosis, a meta-analysis concluded that it may play a role in forecasting progress when comparing before and after MRS results [84].

Functional MRI (fMRI) generates brain activity via a bold oxygen level-dependent (BOLD) signal that measures blood flow and volume shifts. In comparison to controls, AD patients show no or less stimulation of the HC and other MTL structures while doing memory tasks. Activation of the parietal and posterior cingulate sulci suggests some compensation. While offering insight into pathophysiology, fMRI clinical usage is not supported due to limitations such as a low signal-to-noise ratio and the debatable relevance of BOLD as a neuronal activity measure. Variability may be caused by uncontrolled hemodynamic factors [85].

**Figure 10.**
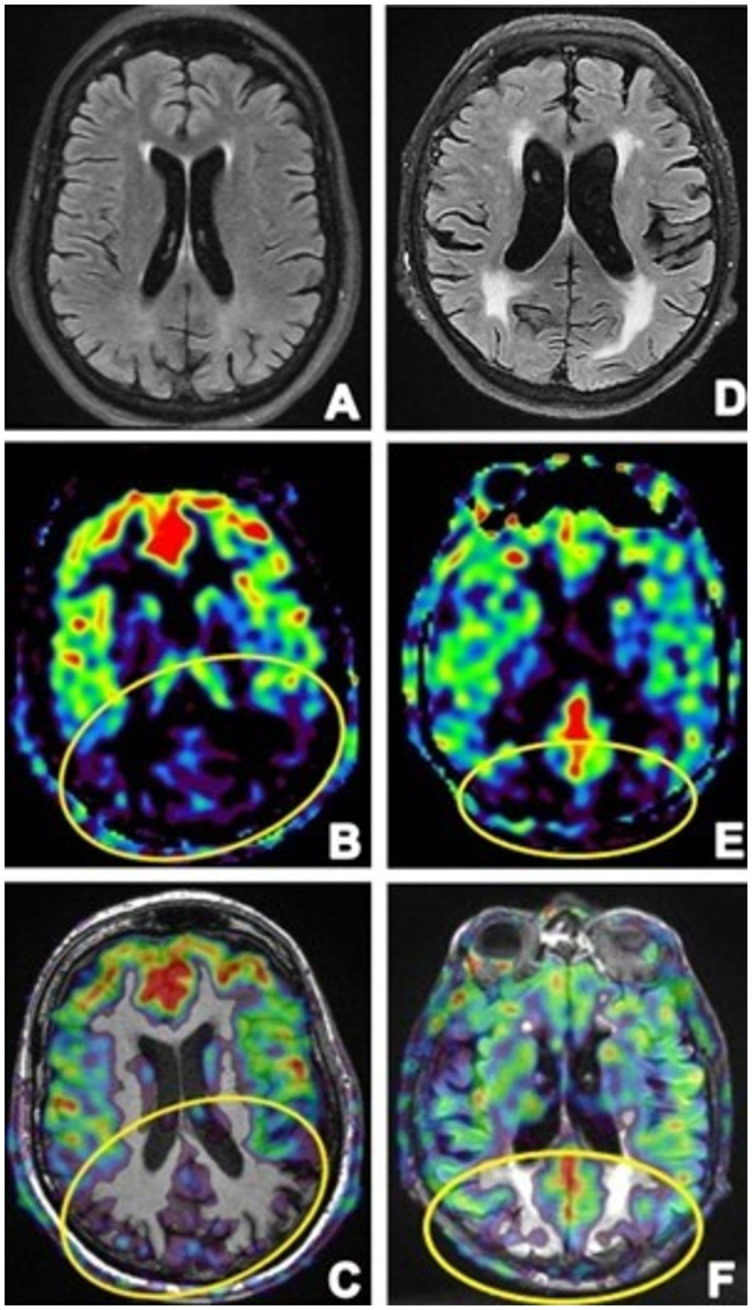
**The images were obtained in a 56-year old female patient with atypical Alzheimer’s disease manifestations (the first column) and a 74-year old male patient with Lewy body dementia** **clinical manifestations (the second column).** The first column: (A) axial FLAIR shows a normal morphological structure of the brain, whereas ASL in (B) shows decreased perfusion in the parietooccipital region, dominantly on the left side. Image (C) shows a fusion of FLAIR (A) and ASL (B) images, for a precise presentation of brain changes. In the second column: (D) axial FLAIR shows moderate atrophy of the frontal and temporal region, while in (E) ASL only shows a mild decrease in perfusion in the parietooccipital region, (F) fusion of FLAIR (D) and ASL (E) image for precise interpretation. Fusion images were made on the General Electronics 3T MRI workstation. ASL: Arterial spin labeling; FLAIR: Three-dimensional T2-weighted fluid-attenuated inversion-recovery imaging; MRI: Magnetic resonance imaging.

Three-dimensional pseudo-continuous ASL provides us with absolute and relative cerebral blood flow (CBF) measures and might be able to identify functional deficits in the brain, similar to FDG-PET and SPECT [86]. Its values have been demonstrated to be comparable with fMRI-BOLD imaging; however, unlike BOLD, ASL offers a quantitative evaluation reflecting brain physiology and neuronal activity [87]. Maps of CBF are often qualitatively assessed with image statistical approaches. Hypoperfusion in the posterior cingulate and precuneus, along with the temporoparietal association cortex, seems to be common AD finding (Figure 10) [88]. Hypoperfusion is commonly identified in bilateral frontal lobes of FTD patients. In comparison to FTD, AD patients had apparent hypoperfusion in the posterior cingulate cortex, and both the sensitivity and specificity in discriminating presenile AD from FTD exceeded 70% [87]. The lower precuneus and cuneus perfusion in LBD has been related to poor overall clinical scores, and the hypoperfusion pattern seems to be comparable to the hypometabolism pattern on FDG-PET [89].

Furthermore, ASL has substantial advantages over PET and SPECT: it is noninvasive and does not expose to ionizing radiation, intravenous contrast agents, or radioactive isotopes; it can be conducted fast with most MRI scanners and repeated quickly. Furthermore, perfusion and structural images could be obtained in the same scan session [86].

The ASL values might change with aging. According to the existing research, younger patients had higher CBF values than seniors. CBF decreases with age and may be caused by reduced cerebral metabolism induced by brain tissue loss. The patient’s age, as well as vascular risk factors, such as carotid disease, should be considered while examining the absolute CBF perfusion values [90].

## Use of MRI—is there a purpose?

Indicated on time, MRI could lead to earlier diagnosis, thus enabling effective treatment and modification of risk factors. Research indicates that a high-quality diet, regular exercise, and a healthier lifestyle can delay the progression of dementia, reduce the chance of developing symptoms, and positively impact MCI [91]. Dietary changes, such as the Asian and Mediterranean diets that are high in unsaturated fats and antioxidants, as well as diets high in vitamins (E, C, B6, D, B12, and folate), fruits and vegetables, accompanied by caloric restriction, have also provided some beneficial results [92].

Clinical trials and therapy evaluation might certainly benefit from neuroimaging. Current studies, mainly considering AD, rely on clinical assessments of cognition and behavior, which vary from day to day and from examiner to examiner. According to Chao et al. [86], ASL-detected hypoperfusion can forecast eventual clinical and cognitive deterioration and may be beneficial in selecting candidates for future AD therapy studies. So far, cholinesterase inhibitors have been mainly used. However, recently the most intensively studied medications include amyloid beta-directed antibodies, such as aducanumab, a medication for early AD or MCI, authorized by FDA. It is the first medication aimed at the underlying AD pathophysiology. According to the results of clinical trials, a reduction in the plaques associated with AD is likely to reduce cognitive and clinical deterioration [93]. An MRI is required to identify ARIA caused by amyloid-modifying drugs. ARIA that could be caused by amyloid beta-directed antibodies include ARIA-E: parenchymal oedema and sulcal effusions, and ARIA-H: microhaemorrhages and localized superficial siderosis. To enable early detection of these side effects, the FDA suggested MRI prior to the application of the initial, 7th, and 12th treatments [7].

Another essential topic is the application of artificial intelligence (AI) algorithms in collaboration with MRI methods. Studies have already shown that employing AI alongside MRI evaluation in achieving precise diagnosis of dementia, including early identification and distinction between different types of dementia, increases diagnostic accuracy. So far, AI has been rarely applied; however, due to its high diagnostic value, it will undoubtedly be used in everyday dementia diagnostic assessment [94].

## Conclusion

MRI has become one of the key diagnostic elements, not only in diagnosis but also in monitoring the therapeutic response of patients with dementia. Semiquantitative scales have been quite useful in the assessment of structural MRI; nevertheless, for even greater precision, they need to be complemented by other advanced techniques. Volumetric analysis and ASL have demonstrated a capacity to improve not only the diagnosis but also the precision in the prognosis of dementia. ASL may be used in cognitive decline prediction and may be effective in selecting candidates for future dementia therapies. However, further research is necessary for even greater usability of the abovementioned MRI techniques.

## References

[1] Álvarez-Linera Prado J, Jiménez-Huete A (2019). Neuroimaging in dementia. Clinical–radiological correlation. Radiología.

[2] Wortmann M (2012). Dementia: a global health priority–highlights from an ADI and World Health Organization report. Alzheimer’s Res Ther.

[3] Jongsiriyanyong S, Limpawattana P (2018). Mild cognitive impairment in clinical practice: a review article. Am J Alzheimers Dis Dementias.

[4] Petersen RC, Lopez O, Armstrong MJ, Getchius TSD, Ganguli M, Gloss D (2018). Practice guideline update summary: mild cognitive impairment: report of the guideline development, dissemination, and implementation subcommittee of the American Academy of Neurology. Neurology.

[5] 2021 Alzheimer’s disease facts and figures. (2021). Alzheimers Dement J Alzheimers Assoc.

[6] Jack CR, Albert M, Knopman DS, McKhann GM, Sperling RA, Carillo M (2011). Introduction to revised criteria for the diagnosis of Alzheimer’s disease: National Institute on Aging and the Alzheimer Association Workgroups on Diagnostic Guidelines for Alzheimer’s disease. Alzheimers Dement J Alzheimers Assoc.

[7] Barakos J, Purcell D, Suhy J, Chalkias S, Burkett P, Grassi CM (2022). Detection and management of amyloid-related imaging abnormalities in patients with Alzheimer’s disease treated with anti-amyloid beta therapy. J Prev Alzheimers Dis.

[8] Shaji KS, Sivakumar PT, Rao GP, Paul N (2018). Clinical Practice Guidelines for management of dementia. Indian J Psychiatry.

[9] Vernooij MW, van Buchem MA. Neuroimaging in dementia. http://www.ncbi.nlm.nih.gov/books/NBK554327..

[10] Young JJ, Lavakumar M, Tampi D, Balachandran S, Tampi RR (2018). Frontotemporal dementia: latest evidence and clinical implications. Ther Adv Psychopharmacol.

[11] Allegri RF (2020). Moving from neurodegenerative dementias, to cognitive proteinopathies, replacing “where” by “what”…. Dement Neuropsychol.

[12] Rodríguez-Gómez JA, Kavanagh E, Engskog-Vlachos P, Engskog MKR, Herrera AJ, Espinosa-Oliva AM (2020). Microglia: agents of the CNS pro-inflammatory response. Cells.

[13] Patel KP, Wymer DT, Bhatia VK, Duara R, Rajadhyaksha CD (2020). Multimodality imaging of dementia: clinical importance and role of integrated anatomic and molecular imaging. RadioGraphics.

[14] Harrison JR, Bhatia S, Tan ZX, Mirza-Davies A, Benkert H, Tax CMW (2020). Imaging Alzheimer’s genetic risk using diffusion MRI: a systematic review. NeuroImage Clin.

[15] Saeed U, Desmarais P, Masellis M (2021). The APOE ɛ4 variant and hippocampal atrophy in Alzheimer’s disease and Lewy body dementia: a systematic review of magnetic resonance imaging studies and therapeutic relevance. Expert Rev Neurother.

[16] Ismail Z, Black SE, Camicioli R, Chertkow H, Herrmann N, Laforce R (2020). Recommendations of the 5th Canadian Consensus Conference on the Diagnosis and treatment of dementia. Alzheimers Dement.

[17] Chertkow H, Feldman HH, Jacova C, Massoud F (2013). Definitions of dementia and predementia states in Alzheimer’s disease and vascular cognitive impairment: consensus from the Canadian conference on diagnosis of dementia. Alzheimers Res Ther.

[18] Oldan JD, Jewells VL, Pieper B, Wong TZ (2021). Complete evaluation of dementia: PET and MRI correlation and diagnosis for the neuroradiologist. AJNR Am J Neuroradiol.

[19] Schmitt T, Rieger JW (2021). Recommendations of choice of head coil and prescan normalize filter depend on region of interest and task. Front Neurosci.

[20] Panman JL, To YY, van der Ende EL, Poos JM, Jiskoot LC, Meeter LHH (2019). Bias introduced by multiple head coils in MRI research: an 8 channel and 32 channel coil comparison. Front Neurosci.

[21] Vernooij MW, Pizzini FB, Schmidt R, Smits M, Yousry TA, Bargallo N (2019). Dementia imaging in clinical practice: a European-wide survey of 193 centres and conclusions by the ESNR working group. Neuroradiology.

[22] Harper L, Bouwman F, Burton EJ, Barkhof F, Scheltens P, O’Brien JT (2017). Patterns of atrophy in pathologically confirmed dementias: a voxelwise analysis. J Neurol Neurosurg Psychiatry.

[23] Van Someren EJW, Oosterman JM, Van Harten B, Vogels RL, Gouw AA, Weinstein HC (2019). Medial temporal lobe atrophy relates more strongly to sleep-wake rhythm fragmentation than to age or any other known risk. Neurobiol Learn Mem.

[24] Elder GJ, Mactier K, Colloby SJ, Watson R, Blamire AM, O’Brien JT (2017). The influence of hippocampal atrophy on the cognitive phenotype of dementia with Lewy bodies. Int J Geriatr Psychiatry.

[25] Huang L, Chen K, Hu X, Guo Q (2020). Differential atrophy in the hippocampal subfield volumes in four types of mild dementia. Front Neurosci.

[26] Fumagalli GG, Basilico P, Arighi A, Bocchetta M, Dick KM, Cash DM (2018). Distinct patterns of brain atrophy in genetic frontotemporal dementia initiative (GENFI) cohort revealed by visual rating scales. Alzheimers Res Ther.

[27] Mak E, Su L, Williams GB, O’Brien JT (2014). Neuroimaging characteristics of dementia with Lewy bodies. Alzheimers Res Ther.

[28] Holden SK, Bettcher BM, Pelak VS (2020). Update on posterior cortical atrophy. Curr Opin Neurol.

[29] Lehmann M, Koedam ELGE, Barnes J, Bartlett JW, Ryan NS, Pijnenburg YAL (2012). Posterior cerebral atrophy in the absence of medial temporal lobe atrophy in pathologically-confirmed Alzheimer’s disease. Neurobiol Aging.

[30] Watson R, Colloby SJ, Blamire AM, Wesnes KA, Wood J, O’Brien JT (2017). Does attentional dysfunction and thalamic atrophy predict decline in dementia with Lewy bodies?. Parkinsonism Relat Disord.

[31] Low A, Mak E, Malpetti M, Chouliaras L, Nicastro N, Su L (2019). Asymmetrical atrophy of thalamic subnuclei in Alzheimer’s disease and amyloid-positive mild cognitive impairment is associated with key clinical features. Alzheimers Dement Amst Neth.

[32] Biel D, Steiger TK, Bunzeck N (2021). Age-related iron accumulation and demyelination in the basal ganglia are closely related to verbal memory and executive functioning. Sci Rep.

[33] Shams S, Martola J, Cavallin L, Granberg T, Shams M, Aspelin P (2015). SWI or T2*: which MRI sequence to use in the detection of cerebral microbleeds? the Karolinska imaging dementia study. AJNR Am J Neuroradiol.

[34] Stojanov D, Aracki-Trenkic A, Vojinovic S, Ljubisavljevic S, Benedeto-Stojanov D, Tasic A (2015). Imaging characteristics of cerebral autosomal dominant arteriopathy with subcortical infarcts and leucoencephalopathy (CADASIL). Bosn J Basic Med Sci.

[35] Haller S, Vernooij MW, Kuijer JPA, Larsson EM, Jäger HR, Barkhof F (2018). Cerebral microbleeds: imaging and clinical significance. Radiology.

[36] Chesebro AG, Amarante E, Lao PJ, Meier IB, Mayeux R, Brickman AM (2021). Automated detection of cerebral microbleeds on T2*-weighted MRI. Sci Rep.

[37] Akoudad S, Wolters FJ, Viswanathan A, de Bruijn RF, van der Lugt A, Hofman A (2016). Association of cerebral microbleeds with cognitive decline and dementia. JAMA Neurol.

[38] Hu HY, Ou YN, Shen XN, Qu Y, Ma YH, Wang ZT (2021). White matter hyperintensities and risks of cognitive impairment and dementia: a systematic review and meta-analysis of 36 prospective studies. Neurosci Biobehav Rev.

[39] Wang J, Zhou Y, He Y, Li Q, Zhang W, Luo Z (2022). Impact of different white matter hyperintensities patterns on cognition: a cross-sectional and longitudinal study. NeuroImage Clin.

[40] Ghaznawi R, Geerlings MI, Jaarsma-Coes MG, Zwartbol MH, Kuijf HJ, van der Graaf Y (2019). The association between lacunes and white matter hyperintensity features on MRI: the SMART-MR study. J Cereb Blood Flow Metab.

[41] Brugulat-Serrat A, Salvadó G, Sudre CH, Grau-Rivera O, Suárez-Calvet M, Falcon C (2020). Patterns of white matter hyperintensities associated with cognition in middle-aged cognitively healthy individuals. Brain Imaging Behav.

[42] Furtner J, Prayer D (2021). Neuroimaging in dementia. Wien Med Wochenschr 1946.

[43] Cannistraro RJ, Badi M, Eidelman BH, Dickson DW, Middlebrooks EH, Meschia JF (2019). CNS small vessel disease. Neurology.

[44] Kalaria RN, Akinyemi R, Ihara M (2016). Stroke injury, cognitive impairment and vascular dementia. Biochim Biophys Acta.

[45] Zhao L, Biesbroek JM, Shi L, Liu W, Kuijf HJ, Chu WW (2018). Strategic infarct location for post-stroke cognitive impairment: a multivariate lesion-symptom mapping study. J Cereb Blood Flow Metab.

[46] Wahlund LO, Westman E, van Westen D, Wallin A, Shams S, Cavallin L (2016). Imaging biomarkers of dementia: recommended visual rating scales with teaching cases. Insights Imaging.

[47] Harper L, Barkhof F, Fox NC, Schott JM (2015). Using visual rating to diagnose dementia: a critical evaluation of MRI atrophy scales. J Neurol Neurosurg Psychiatry.

[48] Scheltens P, Leys D, Barkhof F, Huglo D, Weinstein HC, Vermersch P (1992). Atrophy of medial temporal lobes on MRI in “probable” Alzheimer’s disease and normal ageing: diagnostic value and neuropsychological correlates. J Neurol Neurosurg Psychiatry.

[49] Galton CJ, Gomez-Anson B, Antoun N, Scheltens P, Patterson K, Graves M (2001). Temporal lobe rating scale: application to Alzheimer’s disease and frontotemporal dementia. J Neurol Neurosurg Psychiatry.

[50] Urs R, Potter E, Barker W, Appel J, Loewenstein DA, Zhao W (2009). Visual rating system for assessing magnetic resonance images: a tool in the diagnosis of mild cognitive impairment and Alzheimer disease. J Comput Assist Tomogr.

[51] Duara R, Loewenstein DA, Potter E, Appel J, Greig MT, Urs R (2008). Medial temporal lobe atrophy on MRI scans and the diagnosis of Alzheimer disease. Neurology.

[52] Pasquier F, Leys D, Weerts JG, Mounier-Vehier F, Barkhof F, Scheltens P (1996). Inter- and intraobserver reproducibility of cerebral atrophy assessment on MRI scans with hemispheric infarcts. Eur Neurol.

[53] Park M, Moon WJ (2016). Structural MR imaging in the diagnosis of Alzheimer’s disease and other neurodegenerative dementia: current imaging approach and future perspectives. Korean J Radiol.

[54] O’Donovan J, Watson R, Colloby SJ, Firbank MJ, Burton EJ, Barber R (2013). Does posterior cortical atrophy on MRI discriminate between Alzheimer’s disease, dementia with Lewy bodies, and normal aging?. Int Psychogeriatr.

[55] Scheltens P, Pasquier F, Weerts JG, Barkhof F, Leys D (1997). Qualitative assessment of cerebral atrophy on MRI: inter- and intra-observer reproducibility in dementia and normal aging. Eur Neurol.

[56] Koedam ELGE, Lehmann M, van der Flier WM, Scheltens P, Pijnenburg YAL, Fox N (2011). Visual assessment of posterior atrophy development of a MRI rating scale. Eur Radiol.

[57] Silhan D, Bartos A, Mrzilkova J, Pashkovska O, Ibrahim I, Tintera J (2020). The parietal atrophy score on brain magnetic resonance imaging is a reliable visual scale. Curr Alzheimer Res.

[58] Fazekas F, Chawluk J, Alavi A, Hurtig H, Zimmerman R (1987). MR signal abnormalities at 1.5 T in Alzheimer’s dementia and normal aging. Am J Roentgenol.

[59] Cao Z, Mai Y, Fang W, Lei M, Luo Y, Zhao L (2022). The correlation between white matter hyperintensity burden and regional brain volumetry in patients with Alzheimer’s disease. Front Hum Neurosci.

[60] Claus JJ, Staekenborg SS, Holl DC, Roorda JJ, Schuur J, Koster P (2017). Practical use of visual medial temporal lobe atrophy cut-off scores in Alzheimer’s disease: validation in a large memory clinic population. Eur Radiol.

[61] Wei M, Shi J, Ni J, Zhang X, Li T, Chen Z (2019). A new age-related cutoff of medial temporal atrophy scale on MRI improving the diagnostic accuracy of neurodegeneration due to Alzheimer’s disease in a Chinese population. BMC Geriatr.

[62] Yuan Z, Pan C, Xiao T, Liu M, Zhang W, Jiao B (2019). Multiple visual rating scales based on structural MRI and a novel prediction model combining visual rating scales and age stratification in the diagnosis of Alzheimer’s disease in the Chinese population. Front Neurol.

[63] volBrain: automated MRI brain volumetry system [Internet]. https://volbrain.upv.es/members.php.

[64] FreeSurfer [Internet]. FreeSurfer. https://surfer.nmr.mgh.harvard.edu.

[65] NeuroQuant–Cortechs.ai [Internet]. (2018). https://www.cortechs.ai/products/neuroquant.

[66] Pemberton HG, Goodkin O, Prados F, Das RK, Vos SB, Moggridge J (2021). Automated quantitative MRI volumetry reports support diagnostic interpretation in dementia: a multi-rater, clinical accuracy study. Eur Radiol.

[67] Mårtensson G, Håkansson C, Pereira JB, Palmqvist S, Hansson O, van Westen D (2020). Medial temporal atrophy in preclinical dementia: visual and automated assessment during six year follow-up. NeuroImage Clin.

[68] Duan Y, Lin Y, Rosen D, Du J, He L, Wang Y (2020). Identifying morphological patterns of hippocampal atrophy in patients with mesial temporal lobe epilepsy and Alzheimer disease. Front Neurol.

[69] Wu D, Moghekar A, Shi W, Blitz AM, Mori S (2021). Systematic volumetric analysis predicts response to CSF drainage and outcome to shunt surgery in idiopathic normal pressure hydrocephalus. Eur Radiol.

[70] Ganguli M, Fu B, Snitz BE, Hughes TF, Chang CCH (2013). Mild cognitive impairment: incidence and vascular risk factors in a population-based cohort. Neurology.

[71] Goukasian N, Porat S, Blanken A, Avila D, Zlatev D, Hurtz S (2019). Cognitive correlates of hippocampal atrophy and ventricular enlargement in adults with or without mild cognitive impairment. Dement Geriatr Cogn Disord Extra.

[72] Pyun JM, Park YH, Kim HR, Suh J, Kang MJ, Kim BJ (2017). Posterior atrophy predicts time to dementia in patients with amyloid-positive mild cognitive impairment. Alzheimers Res Ther.

[73] Shiga Y, Miyazawa K, Sato S, Fukushima R, Shibuya S, Sato Y (2004). Diffusion-weighted MRI abnormalities as an early diagnostic marker for Creutzfeldt-Jakob disease. Neurology.

[74] Hermann P, Appleby B, Brandel JP, Caughey B, Collins S, Geschwind M (2021). Biomarkers and diagnostic guidelines for sporadic Creutzfeldt-Jakob disease. Lancet Neurol.

[75] Taoka T, Yasuno F, Morikawa M, Inoue M, Kiuchi K, Kitamura S (2016). Diffusion tensor studies and voxel-based morphometry of the temporal lobe to determine the cognitive prognosis in cases of Alzheimer’s disease and mild cognitive impairment: Do white matter changes precede gray matter changes?. SpringerPlus.

[76] Bergamino M, Keeling EG, Walsh RR, Stokes AM (2021). Systematic assessment of the impact of DTI methodology on fractional anisotropy measures in Alzheimer’s disease. Tomography.

[77] Li Z, Gao H, Zeng P, Jia Y, Kong X, Xu K (2021). Secondary degeneration of white matter after focal sensorimotor cortical ischemic stroke in rats. Front Neurosci.

[78] Shim G, Choi K, Kim D, Suh S, Lee S, Jeong H (2017). Predicting neurocognitive function with hippocampal volumes and DTI metrics in patients with Alzheimer’s dementia and mild cognitive impairment. Brain Behav.

[79] Du J, Zhu H, Yu L, Lu P, Qiu Y, Zhou Y (2021). Multi-dimensional diffusion tensor imaging biomarkers for cognitive decline from the preclinical stage: a study of post-stroke small vessel disease. Front Neurol.

[80] Maul S, Giegling I, Rujescu D (2020). Proton magnetic resonance spectroscopy in common dementias—current status and perspectives. Front Psychiatry.

[81] Sheikh-Bahaei N (2020). MR spectroscopy in Alzheimer’s disease. Biomed Spectrosc Imaging.

[82] Wilson M, Andronesi O, Barker PB, Bartha R, Bizzi A, Bolan PJ (2019). Methodological consensus on clinical proton MRS of the brain: review and recommendations. Magn Reson Med.

[83] Zöllner HJ, Považan M, Hui SCN, Tapper S, Edden RAE, Oeltzschner G (2021). Comparison of different linear-combination modeling algorithms for short-TE proton spectra. NMR Biomed.

[84] Liu H, Zhang D, Lin H, Zhang Q, Zheng L, Zheng Y (2021). Meta-analysis of neurochemical changes estimated via magnetic resonance spectroscopy in mild cognitive impairment and Alzheimer’s disease. Front Aging Neurosci.

[85] Chandra A, Dervenoulas G, Politis M (2019). Magnetic resonance imaging in Alzheimer’s disease and mild cognitive impairment. J Neurolg.

[86] Chao LL, Buckley ST, Kornak J, Schuff N, Madison C, Yaffe K (2010). ASL perfusion MRI predicts cognitive decline and conversion from MCI to dementia. Alzheimer Dis Assoc Disord.

[87] Zhang N, Gordon ML, Goldberg TE (2017). Cerebral blood flow measured by arterial spin labeling MRI at resting state in normal aging and Alzheimer’s disease. Neurosci Biobehav Rev.

[88] Roquet D, Sourty M, Botzung A, Armspach J-P, Blanc F (2016). Brain perfusion in dementia with Lewy bodies and Alzheimer’s disease: an arterial spin labeling MRI study on prodromal and mild dementia stages. Alzheimers Res Ther.

[89] Nedelska Z, Senjem ML, Przybelski SA, Lesnick TG, Lowe VJ, Boeve BF (2018). Regional cortical perfusion on arterial spin labeling MRI in dementia with Lewy bodies: associations with clinical severity, glucose metabolism and tau PET. NeuroImage Clin.

[90] Aracki-Trenkic A, Law-Ye B, Radovanovic Z, Stojanov D, Dormont D, Pyatigorskaya N (2020). ASL perfusion in acute ischemic stroke: the value of CBF in outcome prediction. Clin Neurol Neurosurg.

[91] George EK, Reddy PH (2019). Can healthy diets, regular exercise, and better lifestyle delay the progression of dementia in elderly individuals?. J Alzheimers Dis.

[92] Silva MVF, Loures C de MG, Alves LCV, de Souza LC, Borges KBG, Carvalho M das G (2019). Alzheimer’s disease: risk factors and potentially protective measures. J Biomed Sci.

[93] Research C for DE and. FDA’s decision to approve new treatment for Alzheimer’s disease. FDA [Internet]. (2021 Jun 7). https://www.fda.gov/drugs/news-events-human-drugs/fdas-decision-approve-new-treatment-alzheimers-disease.

[94] Battineni G, Chintalapudi N, Hossain MA, Losco G, Ruocco C, Sagaro GG (2022). Artificial intelligence models in the diagnosis of adult-onset dementia disorders: a review. Bioengineering.

[95] Sotoudeh H, Sarrami AH, Wang JX, Saadatpour Z, Razaei A, Gaddamanugu S (2021). Susceptibility-weighted imaging in neurodegenerative disorders: a review. J Neuroimaging Off J Am Soc Neuroimaging.

